# Intrauterine Fetal Demise, Spontaneous Abortion and Congenital Cytomegalovirus: A Systematic Review of the Incidence and Histopathologic Features

**DOI:** 10.3390/v16101552

**Published:** 2024-09-30

**Authors:** Megan H. Pesch, Jonathan Mowers, Anh Huynh, Mark R. Schleiss

**Affiliations:** 1 Division of Developmental and Behavioral Pediatrics, University of Michigan, Ann Arbor, MI 48109, USA; 2 Division of Pathology, Ascension Hospital Providence, Southfield, MI 48075, USA; jonathanmowers@gmail.com; 3 Department of Pathology, Massachusetts General Hospital, Boston, MA 02114, USA; ahuynh6@mgh.harvard.edu; 4 Division of Pediatric Infectious Diseases, University of Minnesota, Minneapolis, MN 55455, USA; schleiss@umn.edu

**Keywords:** cytomegalovirus, congenital cytomegalovirus, placenta, spontaneous abortion, intrauterine fetal demise, stillbirth

## Abstract

The objective was to review the existing literature reporting on spontaneous abortion (SA) and intrauterine fetal demise (IUFD) associated with cytomegalovirus (CMV) infection. A review using standardized terminology such as ‘intrauterine fetal death’, ‘congenital cytomegalovirus’ and ‘CMV’ was performed using PubMed and Embase (Medline) using the Preferred Reporting Items for Systematic Reviews and Meta-Analyses (PRISMA) methodology. Twenty-one studies met inclusion criteria. CMV was identified as a potential or likely factor in a median of 7.1% of SA or IUFD in study cohorts. Of the studies, 11 used fetal remains, 18 used placenta, 6 used serum, and 1 used post-mortem dried blood spot as specimens for testing for CMV. Features commonly observed were fetal thrombotic vasculopathy, hydrops fetalis and chronic villitis. CMV is frequently noted in studies evaluating viral etiologies of SA or IUFD. Large population-based studies are needed to estimate the incidence of CMV-associated SA or IUFD. CMV and congenital CMV should be included on the differential diagnosis in all cases of SA or IUFD of unknown etiology.

## 1. Introduction

Congenital CMV (cCMV) is a common infection among live-born infants, affecting on average 1 in 208 newborns in higher-income countries and 1 in 70 infants in low-to-middle-income nations [1]. The incidence of cCMV in live born infants is often underappreciated due to the lack of standardized testing protocols and the clinically inapparent nature of most congenital infections [2,3,4]. So too may be the case in CMV-impacted pregnancies that result in intrauterine fetal demise (IUFD) or spontaneous abortion (SA). The lack of standardized histopathologic protocols for examining fetal remains or products of conception (POC) that include CMV testing may result in overlooking this common congenital infection as a factor in pregnancy loss [5]. A better understanding of the contribution of CMV to IUFD and SA is important for better estimates of the economic and social burden of CMV infections in pregnancy.

## 2. Overview of Pathophysiology

CMV can adversely impact a pregnancy both directly though fetal infection and also indirectly through placental damage [6]. Mechanisms by which typical placental growth and function are disrupted include viral proliferation within, and immune-mediated damage of, the placental tissues [6,7,8]. We review basic placental development herein. In humans, the trophoblast cells proliferate and differentiate into two layers: the outer syncytiotrophoblasts and the inner cytotrophoblasts [9]. The syncytiotrophoblast is directly exposed to the maternal blood, covering the highly vascular embryonic placental villi which grow into the wall of the uterus to establish a vascular interface for mother-fetus nutrient and gas exchange [9]. The cytotrophoblast layer which grows during the first and second trimester consists of mononuclear cells and contains the stem cells which develop into the differentiated syncytiotrophoblasts. Cytotrophoblasts are surrounded by placental fibroblasts and macrophages that directly contact the fetal blood vessel. Placenta pericytes also work with the trophoblasts to create the blood-placental barrier which is critical for vascular development [10]. In an ex vivo model, CMV-infected placental pericytes have been found to cause abnormal and reduced tissue perfusion [11].

### 2.1. Impact of CMV on the Placenta

There are a myriad of ways in which CMV has been found to disrupt typical placental function, which we summarize herein and in Table 1. We recommend the following work for an in depth review of the topic [8].

CMV has been found to replicate in trophoblast progenitor stem cells (TBPC) and to impact expression of key regulatory proteins critical for villous growth, differentiation, maturation and function [12,13]. Extravillous trophoblasts (EVTs) infected with CMV down-regulate enzymes and proteins (e.g., matrix metalloproteinase-9, integrin alpha-1/beta-1) that may result in impairment of their ability to invade and anchor in the uterine wall. In turn, uterine vasculature may not remodel sufficiently to support sufficient maternal blood flow to sustain the fetus [14]. Cytotrophoblast and EVTs migration can also be impacted by dysregulation of Wingless (wnt) signaling pathways critical for placentation, and thus be associated with intrauterine growth restriction [15]. CMV infection has been shown to induce apoptosis in cells neighboring infected trophoblasts through tumor necrosis factor alpha secretion [14,16,17]. Inflammatory proteins such as cytokines and chemokines also upregulate tumor necrosis factor alpha, which further promotes inflammation. CMV can also counteract the placental enzymes (e.g., indoleamine 2, 3-dioxygenase) which down-regulate maternal T-cells that normally promote maternal immune tolerance of the fetus, which in turn can result in maternal immune rejection of the fetus [18,19]. This has been hypothesized as one possible mechanism for CMV-associated pregnancy loss and preterm birth [18]. CMV-infected placentas also demonstrate changes that are like those observed in the setting of chronic hypoxia. These changes include fibrinoid deposits, fibrosis, avascular villi and edema that can impair placental function and contribute to fetal injury. Vascular endothelial growth factor and its receptor fms-like tyrosine kinase 1 (Flt1) are up-regulated, and amniotic fluid contains elevated levels of soluble Flt1 (sFlt1), an antiangiogenic protein [20].

Despite the multiple mechanisms of fetal and placental insults that can be associated with cCMV infection, diagnosis following SA or IUFD can be challenging. Examination of the placenta is useful in the diagnosis of placental CMV infection [6]. CMV infection does not have specific placental gross examination findings, but the placenta may be edematous with membranous opacities, similar to other infections [21]. Histologic assessment of the placenta by routine hematoxylin and eosin (H&E) staining identifies characteristic cytomegalic cells with enlarged nuclei and CMV intracellular inclusions [22]. Less specific but common histologic features in placenta CMV infection include hyalinized, avascular villi (which are otherwise seen in vasculopathies) and chronic villitis [6]. The latter may be seen with a variety of placental inflammatory conditions and infections, but is especially common in placental CMV, where plasma cells may also be present [6,22,23]. Immunohistochemistry (IHC) using CMV-specific antibodies is often performed to confirm CMV infected cells in placental tissue sections [24]. Other testing modalities, especially polymerase chain reaction (PCR), may be valuable when there is high clinical suspicion for placental CMV infection [25]; however, a positive placental CMV PCR test is a non-specific screen for fetal infection [26]. PCR can also be performed on non-placental specimens including amniotic fluid, urine and saliva of maternal or neonatal origin to contribute to diagnosis. Infant urine and amniotic fluid CMV PCR have the highest sensitivity and specificity for congenital CMV [27]. Viral culture, transmission electron microscopy and in situ hybridization techniques have largely been replaced by PCR-based technologies [28].

### 2.2. Impact of CMV Infection on a Fetus

The range of outcomes of fetuses impacted by CMV is wide, spanning from in utero fetal demise to the seemingly unaffected infant. The spectrum of outcomes, especially regarding central nervous system (CNS) involvement, likely reflects the timing of infection in gestation. CMV infection during the sensitive period of neurogenesis early in gestation is most consistently associated with CNS sequelae, stemming from neuronal migration disorders, and with long-term neurodevelopmental disability in surviving infants [29]. CMV can invade and replicate in fetal neurons, glia and endothelial cells. Although the exact pathogenesis of CNS injury is unknown, some of the proposed mechanisms include: (1) direct viral infection leading to loss of neural stem cells and brain parenchyma; (2) disrupted proliferation of neural progenitor cells and (3) indirect damage mediated by viral-induced fetal and maternal inflammatory responses [29,30].

### 2.3. Summary and Objectives

In summary, as many as 0.8% of all liveborn infants with cCMV die in early infancy in high-income countries [31]. Estimates of IUFD and SA associated with CMV infection remain unknown. There are likely several factors contributing to this knowledge gap, including low rates of histopathologic examination of POC and fetal remains, as well as lack of a universally accepted standardized testing protocol including CMV on those exams that do occur. Understanding the incidence of CMV-associated SA and IUGR is critical to better understanding the impact of cCMV disease on society [32]. Therefore, the objectives of this study were to: (1) review the existing literature reporting on SA and IUFD associated with CMV infection; and (2) summarize the histopathologic findings on autopsy and placental pathology seen in CMV-associated SA and IUFD.

## 3. Materials and Methods

Table 2 displays the pre-established inclusion and exclusion criteria utilized prior to commencing database searches. Our primary focus was on examining studies sourced from peer-reviewed journals. Initial searches were conducted on 4 November 2023, using Medline (PubMed) and Embase (Elsevier), and a follow-up search was performed on 22 May 2024. The search query incorporated terms such as congenital cytomegalovirus, hearing loss, and newborn screening. We focused on published studies from peer-reviewed journals, excluding conference proceedings, abstracts and commentaries.

**Figure 1:**
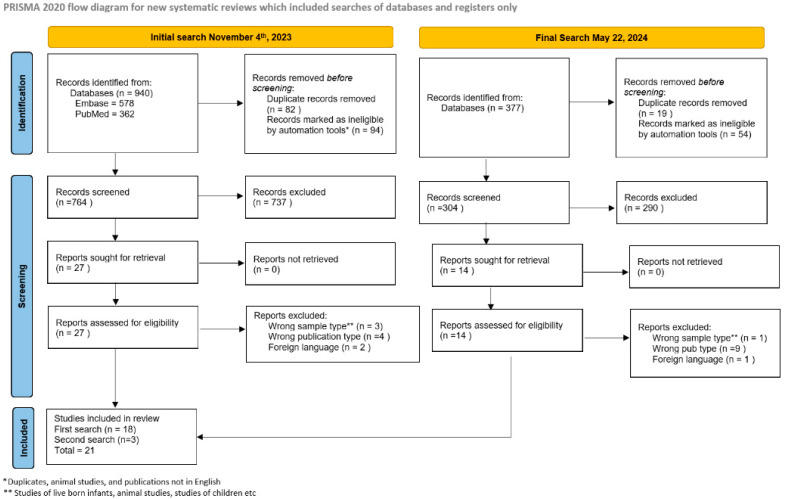
PRISMA diagram from search.

Details of the search terms and constraints applied to each database are outlined in Table 2. A Preferred Reporting Items for Systematic Reviews and Meta-Analyses flowchart is shown in Figure 1. After removal of duplicates, animal studies and publications not in English by automation tools, the screening process resulted in 764 studies for assessment. Title and abstract review excluded 737 studies based on the predetermined criteria, which were reviewed by 2 reviewers. These predetermined criteria were publication in a peer-reviewed journal, English language publication, sample size of >12, in vivo human specimens/subjects and examining the presence of CMV in samples associated with pregnancy loss. This left 27 studies for full-text evaluation, 18 of which met the inclusion criteria. A follow-up search using identical search terminology was conducted of the same databases on 22 May 2024, to determine if any additional studies meeting our inclusion criteria had been published. The updated search yielded 377 additional studies, with 3 meeting final inclusion criteria, thus the total number of included studies from both the original and updated search increased to 21. Data from the 21 studies were extracted and descriptive statistics were calculated, although a formal review of evidence quality and bias risk was not conducted. A review protocol was not prepared. The study was registered with Prospero (ID 566091).

## 4. Results

### 4.1. Summary of Studies

In total, 21 studies were included in the final corpus for the review (Table 3). Of these, cohorts were from Africa (*n* = 1), Asia (*n* = 2), Australia (*n* = 4), Europe (*n* = 7), the Middle East (*n* = 2), North America *(n* = 4) and South America (*n* = 1). In terms of study design, 14 were retrospective in nature, 5 prospective, 1 was cross-sectional, and 1 study was a mixed retrospective and prospective analysis. Twelve studies focused exclusively on IUFD (definitions of which ranged from fetal death after ≥20 weeks to birth), three exclusively investigated SA (conception to <11 weeks), and six examined both conception to <23 weeks). Two studies did not report the gestational ages but referred to the cases as “concepti” (Putland et al.) [33] or “abortions” (Bayati et al.). [34], however referred to these as first trimester losses in their manuscripts; therefore, they were categorized as SAs by our team for this review.

**Figure 2:**
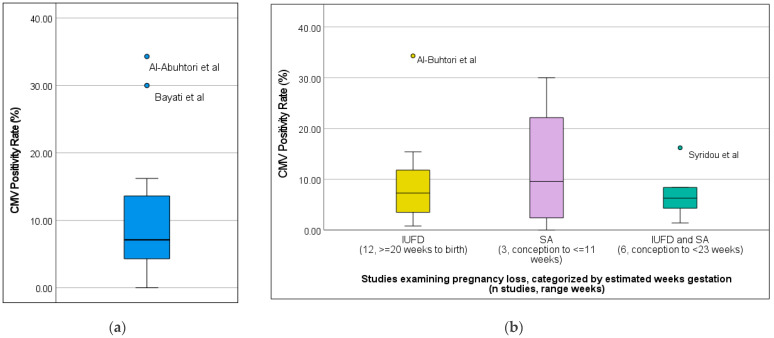
Box plots of CMV positivity rates of tissues from intrauterine fetal demise or spontaneous abortion for studies included in the systematic review. (**a**) CMV Positivity Rate (%) for all entire corpus of studies (*n* = 21). (**b**) CMV Positivity Rate (%) by gestational age of pregnancy loss as categorized by each study (*n* = 21).

Sample sizes ranged from 14 to 2472 pregnancies. Below, we summarize the studies included in our narrative review. Rates of CMV being isolated from specimens of cases in each cohort is henceforth referred to as CMV positivity rate. Note that, even if multiple specimens from a particular case were reported, it is still counted as a single positive case within the cohort of cases. The CMV positivity rate in each study ranged from 0 to 34%, (Median = 7.1%, Q1 3.4%, Q3 14.0%). A box plot of the positivity rates is shown in Figure 2. There were no statistically significant differences between median CMV positivity rates by categorization of pregnancy loss timing (SA vs. IUFD vs. Both) by each study (*p* = 0.62).

### 4.2. CMV-Associated Spontaneous Abortion

Three studies reported rates of CMV infection in SA; rates of CMV infection in ranged from 0% to 30%, with a median of 14.3 [33,34,57].

Yan et al. examined the prevalence of CMV in bodily fluids (blood, urine, cervicovaginal secretions) and placentas in pregnancy and associated pregnancy outcomes in large cohort of 1064 pregnancies, including a control group undergoing dilation and curettage in China [57]. CMV by using PCR or IHC was detected in 63 of 440 people’s bodily fluids early in pregnancy (2011–2012), and of those, 33 (14%) resulted in a first trimester SA [57]. The rates of SA were 47.5% (48/101) and 40.7% (392/963) in women with and without HCMV DNA in cervicovaginal secretion, respectively, and the difference was not statistically significant (*p* > 0.05). Pregnancies with CMV isolated from cervicovaginal fluids were more likely to have CMV isolated from placental specimens after delivery than those without CMV isolated in cervicovaginal fluids (8% vs. 0%).

On the other hand, Putland et al. examined the POC and placenta of “concepti” from SA (the exact gestational age range was not reported) in 350 Australian cases and found no evidence of CMV infection [33] as evidenced by PCR studies. Based on these results, the authors concluded that CMV infection was an unlikely cause of pregnancy loss in the first trimester (although the gestational age of the ‘concepti’ is not described).

Finally, Bayati et al. examined 40 POC from spontaneous abortions (exact gestational age not reported) in a cohort as well as the placenta of 40 live-birth controls for the presence of CMV DNA using IHC [34]. In this Iraqi cohort, they found CMV DNA in 30% and 15% of the SA and live birth groups respectively (*p* < 0.001).

### 4.3. CMV-Associated Intrauterine Fetal Demise

Twelve studies limited their investigation to causes of IUFD, the lower limits of which ranged from GA ≥ 20 to ≥28 weeks estimated gestational age (EGA) [35,36,38,40,41,42,46,48,51,52,54,56]. Rates of CMV infection in cohorts of IUFD specimens ranged from 0.6 to 34% (median = 7.3%, Q1 3.0, Q2 14.1).

In a large Chinese cohort of 44,000 pregnant people, Song et al. identified 2472 (5.6%) who had serological evidence of active CMV infection (positive IgM and low IgG avidity in first trimester) during that time [54]. Thirteen percent (13%) of the pregnancies in the CMV-positive group ended in IUFD (≥28 weeks EGA), versus 214/~41,500 pregnancies (~0.5%) in the CMV-negative group [54]. The authors concluded the relative risk of IUFD in a CMV positive pregnancy (vs. CMV negative) to be 12-fold (RR 12.17; 95% CI 9.43–15.7) [54].

Chow et al. conducted a three-armed study examining 105 placentas from different cohorts: two prospective cohorts of “typical risk” pregnancies and one retrospective cohort from a high-risk pregnancy (with evidence of current or past CMV infection via serologies) [38]. CMV was identified in 4% and 64% of placentas from the low and high risk [35], respectively [38]. Four other studies found rates of CMV positivity in specimens derived from IUFD below 5% [35,42,52,56]. The studies by William et al. [56] and Ahlenius et al. [35] are discussed in detail below in Section 4.5.

In a retrospective study of 130 IUFD, Iwasenko et al. found CMV in 15% of cases [41] using Multiplex PCR. CMV DNA was most often detected in kidney (9%), liver (11%) and less often in placental (5%) specimens [41]. Concordance of infection of the kidney and liver, or kidney, liver and placenta was found in 10% of cases with CMV-isolated in any organ [41]. Fetal thrombotic vasculopathy was the only histopathological abnormality associated with CMV infection (in 60% CMV-infected vs. 28% uninfected IUFD (*p* = 0.010) [41]. Four similar studies reported lower rates of CMV infection in 5–10% of IUFD-derived specimens [40,46,48,51].

### 4.4. Studies Examining IUFD and SA

Six studies reported outcomes of CMV-positivity rates in specimens from IUFD and SA reporting these outcomes together, regardless of gestational age [37,39,47,50,53,58]. The median rate of CMV positivity was 5.5%, range 1.4–16.4, Q1 2.9, Q3 12.3).

Syridou et al. reported on 62 fetal deaths at ≥15 EGA, testing fetal remains and placenta specimens and isolating CMV DNA via molecular assays in 16% of cases compared to only 3% of controls (*p* < 0.05), also noting that this difference increased with gestational age [58].

In an Iranian cohort, Charostad et al. examined 83 cases of IUFD or SA (<24 weeks) and 81 cases of therapeutic TOP (cases in which there was a life threatening anomaly or the life of the mother was at risk), finding CMV in the placenta of both spontaneous (8.4%) and therapeutic (4.9%) abortions [37]. No statistically significant differences were found between these two groups [37].

Studies by Oliveira et al. [50] and Nuovo et al. [47] are discussed in detail in the following section.

### 4.5. Studies Examining IUFD or SA of Infectious Origin

Six studies sought to examine the etiology of SA or IUFD by testing for the presence of several pathogens, including CMV [35,46,47,50,51,56]. Oliviera examined 70 placental samples from SA or IUFG of unknown etiology from a single center in Brazil over a two-year period [50]. Of those, 28 were noted to have villitis, with 5 also having a viral agent isolated (CMV = 3, PVB19 = 1 and HSV 2 = 1) [50]. Two of the three placentas with CMV also had intervillositis; of note, CMV was not isolated using immunohistochemistry with monoclonal anti-CMV antibody (NCL-CMVpp65, Leica Biosystems, Wetzlar, Germany) in any of the placentas without villitis [50].The median GA was 19 at pregnancy loss (range of 8 to 37 weeks); of note, no ultrasound abnormalities were noted in the fetuses [50].

Page et al. conducted a secondary analysis of 512 cases of IUFD in a prospective, multicenter population-based study in the United States, focusing on the 66 (12.9%) cases in which infection was identified as the probable or possible cause of death [51]. Evidence of infection was found in almost all (99%) of placental specimens and 39% of fetal autopsies [51]. In the infection-related cases of IUFD, CMV was found by PCR in 5 (8%) cases. Of the five with CMV, four were noted to have fetal malformations thought to be directly due to fetal infection. It is notable that there were no cases of severe maternal illness prior to the IUFD [51].

Williams et al. conducted a large study of IUFD in an American cohort of 989 cases of SA, IUFD or neonatal deaths considered to be “infective deaths”. 108 were attributed to viral causes [56]. Mortality was attributed to a CMV infection in cases with PCR, serologic and/or histological confirmation, as well as a compatible clinical presentation. Of those, CMV accounted for 20% of total viral-attributable death, or 31.3% (5/16), 33.3% (8/24), 22.7% (5/22), and 8.7% (4/46)of viral-attributable SA, IUFD, neonatal and post-neonatal death respectively [56] CMV-isolation in any of the specimens was associated with a global loss rate of 3.1 (95% CI, 1.7–4.4) per 100,000 births [56].

In another study, a Swedish cohort of 66 specimens of IUFD (>25 weeks) found 7 cases which appeared to be related to viral infection [35]. CMV was isolated by culture in 3 of 7 cases in which it was also thought to be the possible or probable cause of fetal demise [35].

Nuovo et al. examined 21 consecutive cases of SA or “perinatal death” (4 cases SA, 12 cases third trimester IUFD, 5 cases or early infant demise within 48 h of birth) for an infectious agent, as well samples from controls. Infection was noted in 16 of 21 cases, including a single case of CMV [47]. CMV DNA and protein were predominantly localized in the Hofbauer and endothelial cells of the placenta, and in the spleen and lung of the fetal remains [47]. No infectious agents were identified in the control placentas and POC [47].

Similarly, Nasyrov et al. examined placental specimens in cases of suspected infectious IUFD and controls, noting damaged capillaries in the stem villi and foci of “fine clumpy and dust like basophilic disintegration in the villous stroma surrounding them” [46]. They noted narrowed to completely “blocked” lumens by fibrin [46]. Mucoid and fibrinoid edema with foci of fibrin necrosis were also detected in the villi [46]. Poor vascularization of the terminal villi in the IUFD group was also noted, with only 25% with capillaries present along the villous periphery forming a vasculo-syncytial membrane [46]. Immunohistochemical study of 10 IUFD placental specimens identified either HSV or CMV in 9 of 10 cases. Of the 10 placentas, both HSV and CMV antigens were identified in 3, HSV only in 4 cases and CMV only in two cases. TNF antibodies were seen in all chorionic villi. No cases of TNF antigens were found in control cases [46].

## 5. Discussion

This systematic review identified 21 studies examining the occurrence of cCMV in cohorts of pregnancies ending in SA and/or IUFD. Rates of CMV-positive specimens in each study ranged from 0 to 34 from%, (Median 7.1%, Q1 3.4%, Q3 14.0%). In general, rates of CMV-infected specimens in studies were highest in studies examining the prevalence of CMV-infected tissues among those with a cause of SA or IUFD thought to be of infectious origin. Few studies found no cases of CMV-associated SA or IUFD. It is important to note that the studies in this review did not seek to ascertain the population level incidence of SA or IUFD due to CMV or in CMV-impacted pregnancies. Future research should consider the contribution of regional CMV seroprevalence to the rates of CMV positivity among SA and IUFD derived specimens. Given that many pregnancies ending in SA or IUFD are not evaluated for CMV infection due to lack of standardized evaluations, it is likely that these studies underestimate the true prevalence of CMV-associated SA or IUFD in the general population. CMV-infection is an important and under-recognized contributor to SA and IUFD.

There were several studies in our cohort that reported outlying rates of CMV positivity within their cohorts. Putland et al. found no cases of CMV positivity in a cohort of 350 POC from SAs in Australia in 1990 [33], as opposed to other Australian studies which found 5–15% CMV positivity in retrospective cohorts [38,40,41]. It may be that the seropositivity in the area of Australia from which the Putland et al. cohort was derived from was lower in the late 1980s when the study data were collected, as opposed the seropositivity in 2002–2006 when data from the other studies were collected. It may also be that the Putland et al. samples were from a very early gestational age (exact gestational age not reported in the publication, although referred to as “first trimester” and “concepti”). The transmission rate of CMV to embryo/fetus is known to increase with gestational age. It may be that very early pregnancy losses may be less likely to be associated with CMV than later-term losses. Another hypothesis is that the multiple dilution steps used in the Putland et al. PCR could have resulted in a lower sensitivity assay than what was used over a decade later.

On the other end of the spectrum of outliers, Al-Buhtori et al. [36] and Bayati et al. [34] found rates of CMV positivity of 34% and 30%, respectively, in cohorts from the United Kingdom and Iraq. It is possible that the CMV positivity rate in the Al-Buhtori et al. study was enriched relative to other studies in our review due to sample selection bias, as only fetuses with an unknown cause of demise were included in the cohort [36]. In the Bayati et al. cohort, in which 30% of pregnancy loss samples had CMV identified in specimens, so did 15% of controls [34]. This is not surprising given the high seroprevalence in the Middle East in general. Therefore, while CMV was identified in 30% of cases, it is possible that the rate of CMV attributable pregnancy losses in that group was much lower, closer to 15%.

The American College of Obstetricians and Gynecologists recommends that an evaluation of intrauterine fetal demise include fetal autopsy, gross and histologic examination of the placenta, umbilical cord, and membranes and genetic evaluation [59]. In the study by Page et al. which examined 512 cases of IUFD, placental pathology and fetal autopsy were useful in confirming or excluding a potential cause of intrauterine fetal demise in 64.6% and 42.4% of cases, respectively [51]. The utility of additional clinical testing, which can include but is not limited to karyotype, serologies, fetal-maternal hemorrhage or testing for antiphospholipid antibodies, varied by clinical presentation.

In a different study by Miller et al. which examined 144 cases of IUFD, clinical and laboratory data alone only identified a cause of death in 24% of cases [60]. Placental examination improved this rate to 61%, and the addition of autopsy increased the rate to 74% of cases having an identifiable probable cause of death [60]. CMV has been observed in an array of fetal organs, including commonly in pancreas, lungs, kidneys, liver, brain and heart, with the liver and kidney being reported as the two most frequently infected organs in one study [41]. However, not every organ may be affected in every case. Therefore, a standardized approach to autopsy of the fetus that includes CMV testing may be helpful in obtaining diagnostic clarity [60].

Unfortunately, perinatal autopsy rates have been declining despite its diagnostic utility [61]. Less invasive alternative methods of post-mortem evaluation may be more acceptable to bereaved parents, while also being sufficient to evaluation for congenital CMV. These include needle biopsy of organs followed by IHC or qPCR, brain imaging to evaluate for calcifications or disorders of neuronal migration, or histology of the placenta alone.

Placental pathology can provide information to guide clinical management for both mothers and newborns. For the latter, timely communication of immediately treatable conditions such as hematogenous infections (e.g., CMV, *Listeria monocytogenes*), chorioamnionitis, candida funisitis or metabolic storage diseases, can lead to lifesaving interventions [5]. In the case of CMV, the placenta can serve as both a reservoir for vertical transmission to the fetus or as a site of protection that prevents the fetus from viral infection [7]. Placental pathology may be especially important in the diagnosis of cCMV insofar as CMV infections in pregnant women are often clinically mild or asymptomatic, there is a lack of routine CMV serological screening in pregnancy, and even when available, serologic data can be difficult to interpret and cannot reliably predict fetal infection [62,63].

CMV-infected placentas commonly demonstrate villitis, which may be lymphocytic or plasmacytic. Other findings may include villous fibrosis and mineralization, plasma cell infiltrates, hyalinized villi, and Hofbauer cell hyperplasia (Table 1). Viral inclusions can sometimes be identified in the nucleus and cytoplasm of placental endothelial and stromal cells, but the finding of plasma cells and hemosiderin in chorionic villi is strongly associated with CMV infection, even absent the finding of inclusions. Viral inclusions appear to be more abundant in the second trimester than the third trimester [64]. CMV can be isolated from almost any fetal organ, although it does not always infect all organs in every case. In the study by Uenaka et al., the three most frequently reported features were the presence of CMV-positive cells, intranuclear inclusions and chronic villitis [65]. However, it is important to note that detection of CMV in the placenta does not always correlate to fetal infection and vice versa [6].

This study is not without limitations. It is important to note that the incidence of CMV-associated SA or IUFD from individual study cohorts cannot be generalized to the greater population due to ascertainment bias. Many pregnancies ending in SA or IUFD do not undergo histopathologic examination for a variety of reasons (e.g., family declines, early SA at home, cultural practices, lower public health resource area). As such, results should be interpreted with caution. Only studies meeting our inclusion criteria were reviewed, which may have led to the omission of some of the literature. Furthermore, countries with low seroprevalence rates of CMV were disproportionately represented in this review; therefore, results may not be applicable to high seroprevalence areas.

## 6. Conclusions

Cytomegalovirus is an important and under-recognized contributor to SA and IUFD. Placental and fetal histopathology can be helpful in making a diagnosis, as histologic findings of CMV infection can be non-specific and present in many ways across tissues and organs. The importance of both placental and fetal examinations cannot be understated and should be considered in the evaluation of any SA or IUFD. However, in circumstances where bereaved parents may opt for less-invasive fetal examinations, the use of ancillary testing such as molecular methods to detect CMV in fetal biopsy specimens, radiographic findings, serologic and microbiological studies, in addition to placental examination for CMV alone, may offer much-needed information to render the diagnosis of congenital CMV.

## Figures and Tables

**Table 1:** Histopathologic evaluation of specimens in the case of IUFD or SA.

Specimen	Finding	Potential Mechanisms and Comments
Placenta	Viral cytopathic effect CMV viral inclusions CMV IHC/PCR	Viral infection of placental cells
	Endothelial damage RBC extravasation Hemosiderin deposition	CMV-related endothelial damage
	Vasculopathy Villous stromal vascular karyorrhexis/hemorrhage Villous vascular proliferation Avascular villi Villous fibrosis	Sequelae of vascular damage
	Gross edema and membranous opacityChronic villitis Plasma cells * Lymphocytes * Histiocytes	Maternal inflammatory response to placental CMV infection
Fetal remains	Viral cytopathic effect CMV viral inclusions (H&E) CMV IHC/PCR	Viral infection of fetal cells (pancreas, lungs, kidneys, liver, brain, heart, etc.)
	Hydrops fetalisExtramedullary hematopoiesisPetechial hemorrhage	Fetal hypoxia
	Intrauterine growth restriction	Placental insufficiency Fetal hypoxiaTumor Necrosis Factor α-mediated trophoblast apoptosis

* Lymphoplasmacytic chronic villitis is the most identified placental histologic abnormality. Abbreviations: H&E, hematoxylin and eosin; IHC, immunohistochemistry; PCR, polymerase chain reaction.

**Table 2:** Search terms and methods used in the review.

Search No.	Search Terms
#1	Cytomegalovirus [Mesh]
#2	Cytomegalovirus [All fields]
#3	“Congenital cytomegalovirus” [All fields] OR (congenital [All fields] AND cytomegalovirus [All fields])
#4	“Congenital anomalies, virology” (Mesh) OR “congenital anomalies, virology” [All fields]
#5	“Congenital cytomegalovirus disease” [All fields]
#6	“cCMV” [All fields] OR “CMV” [All fields]
#7	“Infections, viral diseases” (Mesh) OR “infections, viral diseases” [All fields]
#8	#1 OR #2 OR #3 OR #4 OR #5 OR #6
#9	Stillbirth [Mesh] OR Stillbirth [All fields] OR “Fetal death” [Mesh] OR “Fetal death” [All fields] OR “Pregnancy complications, pathology” [All fields] OR “Pregnancy complications, pathology” [All fields] OR “Adverse Birth Outcomes” (Mesh) OR “Adverse Birth Outcomes” (All fields) OR “Perinatal death” (Mesh) OR “Perinatal death” (All fields)
#10	#7 AND #8
#11	“Placenta, pathology” [Mesh] OR “Placenta, pathology” [All fields] OR “Placenta, microbiology” [Mesh] OR “Placenta, microbiology” [All fields] OR “Placenta, anatomy and histology” [Mesh] OR “Placenta, anatomy and histology” [All fields] OR “Fetus, pathology” [Mesh] OR “Fetus, pathology” [All fields] OR “Fetus, microbiology” [Mesh] OR “Fetus, microbiology” [All fields] OR “Fetus, anatomy and histology” [Mesh] OR “Fetus, anatomy and histology” [All fields]
#12	#8 AND #11
#13	“Animals” [Mesh] NOT “Humans” [Mesh]
#14	“Comment” [Publication Type] OR “Letter” [Publication Type] OR “Editorial” [Publication Type] OR “Case Reports” [Publication Type] OR “Clinical Trial, Phase I” [Publication Type] OR “case study” [Title] OR “case studies” [Title] OR “case report” [Title] OR “case reports” [Title] OR “case series” [Title]
#15	(#8 OR #12) NOT (#13 OR #14)

**Table 3:** Characteristics of studies of cytomegalovirus-associated spontaneous abortion and intrauterine fetal demise included in the scoping review (*n* = 21).

Study	Country	Study Design	Years	Cohort	IUFD or SA or Both	Definition (Weeks)	Specimens	CMV-Associated SA, IUFD, TOP *n* (%)	Results Summarized
Ahlenius et al., 1995 [35]	Sweden	Retrospective	1987–1989	66 cases of IUFD	IUFD	>25	Fetus Placenta Serum Amniotic fluid	3 (4.5)	Of the 7 cases in which viral infection was diagnosed, CMV was isolated by culture and thought to be a possible or probable cause of death in three cases (42.8%)
Al-Buhtori et al., 2011 [36]	United Kingdom and Australia	Retrospective	1997	73 cases of IUFD with unknown cause of death, 27 cases of TOP as a control group	IUFD	≥20	Fetus Placenta	25 (34.3)	Using PCR, CMV was found in one or more tissue samples from 25/73 cases (34%) by IHC. CMV was not found in any of the TOP samples.
Bayati et al., 2017 [34]	Iraq	Retrospective	NR	40 SA and 40 live born controls	SA	NR	Placenta	15 (30.0)	Using IHC, CMV DNA was isolated in 30% and 15% of the SA and live birth groups respectively (*p* < 0.001).
Charostad et al., 2020 [37]	Iran	Retrospective	2014–2015	83 cases of IUFD or SA and 81 cases of TOP	Both and TOP	<24	Placenta POC	7 (8.4)	CMV was identified by PCR for human β-globin gene using specific primers in the placenta of both spontaneous (8.4%) and therapeutic (4.9%) abortions. No statistically significant differences were found between these two groups.
Chow et al., 2006 [38]	Australia	Prospective and retrospective cohorts	2002–2005	105 placentas from three cohorts. Two prospective cohorts of typical risk pregnancies, one retrospective cohort of women/fetuses with CMV seroconversion in first trimester	IUFD	28–32	Placenta Amniotic fluid	5 (4.7)	CMV identified in 4% and 64% of placentas from low and high-risk groups, respectively, via IHC. Of the 11 pregnancies in the high-risk group, 7 resulted in SA, TOP or IUFD. Of the 7 infants with CMV isolated in placental tissue only 2 were live born.
Grammatikopoulous et al., 2012 [39]	Greece	Retrospective	Not available	143 cases	Both	≥11	Fetus Placenta	2 (1.4)	2 of the 143 fetuses that were examined were found to be CMV positive by PCR. Acute chorioamnionitis was seen in 86/142 examined fetuses including the two with CMV.
Howard et al., 2009 [40]	Australia	Retrospective	2005–2006	107 cases of IUFD	IUFD	≥20	Post-mortem DBS	10 (9.3)	Of the 107 IUFD, 10 (9%) were CMV positive as assessed by PCR (amplification of the gp58 gene). The rate of CMV infection did not differ between early (8%) and late IUFD (9%).
Iwasenko et al., 2011 [41]	Australia	Retrospective	2005–2006	130 cases of IUFD	IUFD	>20	Fetus Placenta	20 (15.4)	CMV was detected in 15% of cases. CMV DNA was detected in kidney (9%), liver (11%), and placenta (5%) specimens, with 75% of infections confirmed by immunohistochemistry and by Multiplex PCR. Fetal thrombotic vasculopathy was the only histopathological abnormality associated with CMV infection (in 60% CMV-infected vs. 28% uninfected IUFD (*p* = 0.010).
Kidron et al., 2009 [42]	Israel	Retrospective	1994–2005	120 cases of IUFD	IUFD	≥23	Fetus Placenta	2 (1.5)	Fetal abnormality was cited as the cause of death in 14/ losses; 2/14 of the causes of death from infection were from cCMV (24 and 30 weeks) as established by standard protocols [43,44,45].
Nasyrov et al., 2020 [46]	Russia	Retrospective	2010–2018	20 cases of IUFD and 10 live born controls	IUFD	≥27	Fetus Placenta	2 (10.0)	2 cases of CMV in 10 samples from IUFD were identified with IHC. CMV antigen detected in the walls of capillaries and in placental villous stroma.
Nuovo et al., 2005 [47]	USA	Prospective	Not reported	21 cases of SA, IUFD or early neonatal death (within 48 h) and 10 TOP controls	Both and early neonatal death	≥17	POC Fetus Placenta	1 (4.8)	One of the 21 cases of pregnancy loss was positive for CMV by in situ hybridization and IHC. 10/21 had Coxsackie virus isolated.
Odendaal et al., 2019 [48]	South Africa	Prospective cohort	2007–2015	14 cases of IUFD	IUFD	20- < 22 weeks	Fetus Placenta	1 (7.1)	CMV identified in 1 of the 14 cases with specimen available for testing by standard protocol [49]. Diffuse chronic villitis was seen on placental pathology.
Oliveira et al., 2019 [50]	Brazil	Cross-sectional	2013–2015	70 placenta samples from SA or IUFD	Both	SA ≤ 20 IUFD > 20	Placenta	3 (4.3)	Using IHC 3/70 samples were positive for CMV: three placentas with villitis, 2 with intervillitis. Mean GA 19 weeks. No ultrasound abnormalities detected.
Page et al., 2019 [51]	USA	Multisite retrospective review	2006–2008	512 IUFD, 66 of which with infectious etiology	IUFD	≥20	Fetus Placenta Amniotic sac	5 (7.5)	8% of IUFD with infectious etiology attributed to CMV. Note that not all specimens were tested for CMV by viral culture or PCR (e.g., only 8% of the cohort had maternal serologies, and CMV only noted on 6 placental pathology or fetal autopsy reports, 5 cases of which were noted to be possible or probably cause of fetal demise).
Petersson et al., 2002 [52]	Sweden	Prospective	1998–1999	188 cases	IUFD	≥22	Serum Amniotic fluid PlacentaFetus	1 (0.6)	91% of cases of IUFD had an established cause. CMV was established to be the cause of one fetal demise.
Plotzker et al., 2022 [53]	USA	Retrospective	2020	16 IUFD in the setting of maternal COVID-19 infection	Both	≥17	Serum Placenta	1 (6.3)	One of the 16 cases of IUFD in the setting of maternal COVID-19 was found to be secondary to a primary CMV co-infection.
Putland et al., 1990 [33]	Australia	Retrospective	NR	350 POC from SA	SA	NR	POC	0 (0)	No CMV DNA was detected in 350 SA samples tested by PCR, or in 36 viral cultures performed.
Song et al., 2022 [54]	China	Prospective cohort	2013–2019	>44,000 pregnant women, 2472 with CMV IgM+/ low IgG avidity in first trimester, 540 total cases ended with IUFD	IUFD	≥28	Maternal serologies	326 (13.6)	Maternal CMV serology group (IgM+): 326/2472 (13%) pregnancies ended with IUFD (86.6 per 1000 pregnancies) vs. 214/~41,500 pregnancies (7.8 per 1000 pregnancies) in the group who were IgM negative(RR 12.17; 95% CI 9.43–15.71).
Syridou et al., 2008 [55]	Greece	Prospective	2004–2005	62 fetal deaths and 35 controls	Both	≥15	Fetus Placenta	10 (16.2)	IUFD/SA and control groups differed in the detection rate of CMV DNA by PCR (16% and 3%, respectively; *p* = 0.047), which was more pronounced in a gestational age >20 weeks (*p* = 0.03).
Williams et al., 2013 [56]	USA	Retrospective database review	1988–2008	>8000 IUFD over a 21-year period, 989 of which were considered definitively involving infection as etiology, 412 of which were tested for CMV	IUFD and early neonatal death	≥20	Fetus Placenta Serum	25 (2.5)	10/247 late fetal losses (20–23 weeks GA) and 15/165 deaths > 23- < birth from infection were likely from CMV, as ascertained by “virologic investigations” that were standard at the time of the fetus’ demise. Representing 2.5% of all losses, or 6.1% from infectious etiology
Yan et al., 2015 [57]	China	Retrospective	2011–2012	1064 pregnancies undergoing D&C, 440 SA, 624 TOP	SA	7–11	Cervicovaginal fluids POC Placenta Serologies	33 (14.3)	Of 63/440 with CMV DNA isolated by PCR or IHC in the first trimester, 33 ended SA. CMV detected in villi or decidua in 3/63.

Abbreviations. CMV, cytomegalovirus; IUFD, intrauterine fetal death; SA, spontaneous abortion; TOP, termination of pregnancy; GA, gestational age; NR, not reported; POC, products of conception.

## Data Availability

Data is contained within the article.
